# Black liquor and the hangover effect: fish assemblage recovery dynamics following a pulse disturbance

**DOI:** 10.1002/ece3.1530

**Published:** 2015-05-25

**Authors:** Kyle R Piller, Aaron D Geheber

**Affiliations:** 1 Department of Biological Sciences, Southeastern Louisiana UniversityHammond, Louisiana, 70402; 2 Department of Biology, University of OklahomaNorman, Oklahoma, 73019

**Keywords:** Community structure, fish kill, Pearl River, spill

## Abstract

Anthropogenic perturbations impact aquatic systems causing wide-ranging responses, from assemblage restructuring to assemblage recovery. Previous studies indicate the duration and intensity of disturbances play a role in the dynamics of assemblage recovery. In August 2011, the Pearl River, United States, was subjected to a weak black liquor spill from a paper mill which resulted in substantial loss of fish in a large stretch of the main channel. We quantified resilience and recovery of fish assemblage structure in the impacted area following the event. We compared downstream (impacted) assemblages to upstream (unimpacted) assemblages to determine initial impacts on structure. Additionally, we incorporated historic fish collections (1988–2011) to examine impacts on assemblage structure across broad temporal scales. Based on NMDS, upstream and downstream sites generally showed similar assemblage structure across sample periods with the exception of the 2 months postdischarge, where upstream and downstream sites visually differed. Multivariate analysis of variance (PERMANOVA) indicated significant seasonal variation among samples, but found no significant interaction between impacted and unimpacted assemblages following the discharge event. However, multivariate dispersion (MVDISP) showed greater variance among assemblage structure following the discharge event. These results suggest that 2 months following the disturbance represent a time period of stochasticity in regard to assemblage structure dynamics, and this was followed by rapid recovery. We term this dynamic the “hangover effect” as it represents the time frame from the cessation of the perturbation to the assemblage's return to predisturbance conditions. The availability and proximity of tributaries and upstream refugia, which were not affected by the disturbance, as well as the rapid recovery of abiotic parameters likely played a substantial role in assemblage recovery. This study not only demonstrates rapid recovery in an aquatic system, but further demonstrates the value of continuous, long-term, data collections which enhance our understanding of assemblage dynamics.

## Introduction

**Figure 1:**
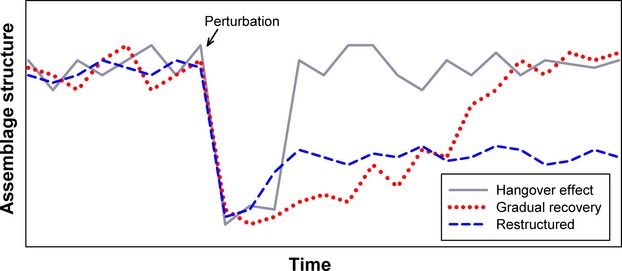
Hypothetical depiction of three modes of recovery in aquatic systems. Gradual recovery refers to assemblage structure response that returns to predisturbance levels over a long period of time. A restructured mode refers to the change in assemblage structure to a novel state. Finally, the hangover effect refers to the short-term, time-lagged response for which an assemblage rapidly returns to predisturbance conditions.

Pollution of aquatic systems through industrial, municipal, and/or agricultural discharge is well known to negatively impact aquatic communities (Detenbeck et al. 1992; Ensign et al. 1997; Ryon 2011). These types of disturbances are referred to as “pulse” disturbances (Bender et al. 1984), as they rapidly impact species numbers, but are followed by a return to an equilibrium state. Pulse disturbances are relatively common in freshwater systems. In some cases, fish assemblage recovery following these types of perturbations has occurred relatively quickly (Meffe and Sheldon 1990; Peterson and Bayley 1993; Albanese et al. 2009), whereas in other situations, it has taken years to return to preperturbation conditions (Detenbeck et al. 1992). Different species recovery dynamics, including the pattern and rate of recovery (Fig.1), have been noted for different perturbation types due to differences in specific life-history traits, the availability and proximity of refugia, and duration and magnitude of the perturbation (Yount and Niemi 1990; Detenbeck et al. 1992). In most cases, however, the dynamics of assemblage recovery (i.e., resiliency) are unknown, particularly in terms of the chronology of species recolonization and attainment of a stable assemblage. Often, this is because studies are limited by the amount of comprehensive historic data describing preperturbation species distributions and abundances for the organisms in the impacted areas (Geheber and Piller 2012).

Quantifying assemblage recovery following environmental perturbations is important and can lead to a better assessment and understanding of the severity and long-term impacts of such events. This is especially important because of potential differences in assemblage resilience, which are dependent on the type, scale, and timing of a disturbance (Ross 2013). As defined by Ross (2013), resilience is the ability of an assemblage to recover following a perturbation event once environmental conditions become more favorable. Under favorable conditions, recovery may occur via several mechanisms, including the return of displaced individuals to repopulate an area after conditions have improved, accelerated production of new individuals, and or recolonization of the disturbed area from surrounding (i.e., unaffected) refugia populations (Ross et al. 1985; Fausch and Bramblett 1991). Although recovery is often measured at the assemblage level, the potential loss of individual species (i.e., rare species) even after assemblage recovery should not be overlooked (Albanese et al. 2009). Therefore, it has become increasingly important for studies that aim to measure assemblage resilience and recovery to also identify population level responses of focal taxa following perturbation events.

Forestry and logging practices represent one of most prominent industries along the Gulf Coastal Plain and generate millions of dollars for local economies (Ward et al. 2005). The Pearl River, in the central Gulf Coastal Plain, originates in central Mississippi and flows in a southwesterly direction toward its mouth in Mississippi Sound. The lower third of the river forms the border between Louisiana and Mississippi. Approximately fifty percent of the Pearl River basin is forested, and logging and timber processing occur primarily in the lower half of the basin (Ward et al. 2005). One of the larger timber processing plants, a paper mill and box plant, occurs in Bogalusa, Washington Parish, Louisiana, and has existed there for more than a century (Curtis 1973). The paper mill discharges waste water into secondary treatment ponds adjacent to the Pearl River and then directly into the main channel of the river. In August 2011, between 25 and 75 million gallons of an industrial by-product known as “weak black liquor” was inadvertently released into the Pearl River over several days near Bogalusa, Louisiana. Weak black liquor is a by-product of the paper making process and consists of lignins, tannins, resin acids, fatty acids, and inorganic chemicals including sulfur and chlorinated compounds, and it typically has a high biological oxygen demand (Ali and Sreekrishnan 2001). As a result of a plugged evaporator, a large volume of untreated weak black liquor was inadvertently discharged into the wastewater sewers, then to treatment ponds, and finally into the Pearl River causing a reduction of dissolved oxygen in the river, resulting in a loss of fish and freshwater mussels in a 40+ km stretch of the river main channel downstream of the mill discharge site. Fish kill estimates by state and federal natural resource agencies indicated a loss of more than 160,000 fishes as a result of the weak black liquor discharge (LDWF 2011). Furthermore, subsequent sampling of abiotic components, including pH, conductivity, dissolved oxygen, by these agencies indicated that the Pearl River returned to normal levels within only a few days of the event (LDWF 2011).

The Pearl River basin harbors approximately 119 different species of freshwater fishes and is relatively high in species richness compared to other rivers of similar size in eastern Louisiana and southern Mississippi (Ross 2001). The ichthyofauna of the Pearl River basin has been studied for more than half of a century (Gunning and Suttkus 1985; Gunning and Suttkus 1991; Piller et al. 2004; Tipton et al. 2004; Stewart et al. 2005; Geheber and Piller 2012) making the ichthyofauna of the Pearl River basin one of most well-known fish assemblages in the southeastern United States. The most recent work indicates despite reductions in overall diversity over a 22-year period (1988–2009), the last five study years have remained relatively stable in terms of species richness and abundance in middle and lower portions of the Pearl River (Geheber and Piller 2012).

Although historic aspects of fish assemblage change in the basin have been well studied, nothing is known in regard to how the fish assemblage responds to acute, short-term perturbations such as the weak black liquor spill in 2011. This study utilized historic museum data on species distributions and abundances in the Pearl River, in conjunction with comprehensive contemporary fieldwork, to investigate the dynamics of assemblage recovery in an environmentally disturbed system. Our objectives were as follows: (1) to examine the immediate impacts of a pulsed perturbation on a freshwater riverine fish assemblage; (2) to assess whether the fish assemblage recovered (i.e., assemblage structure returned to that of preperturbation historic communities) following the perturbation; and (3) to quantify the temporal scale necessary for assemblage recovery following the perturbation.

## Materials and Methods

### Assemblage collections

**Figure 2:**
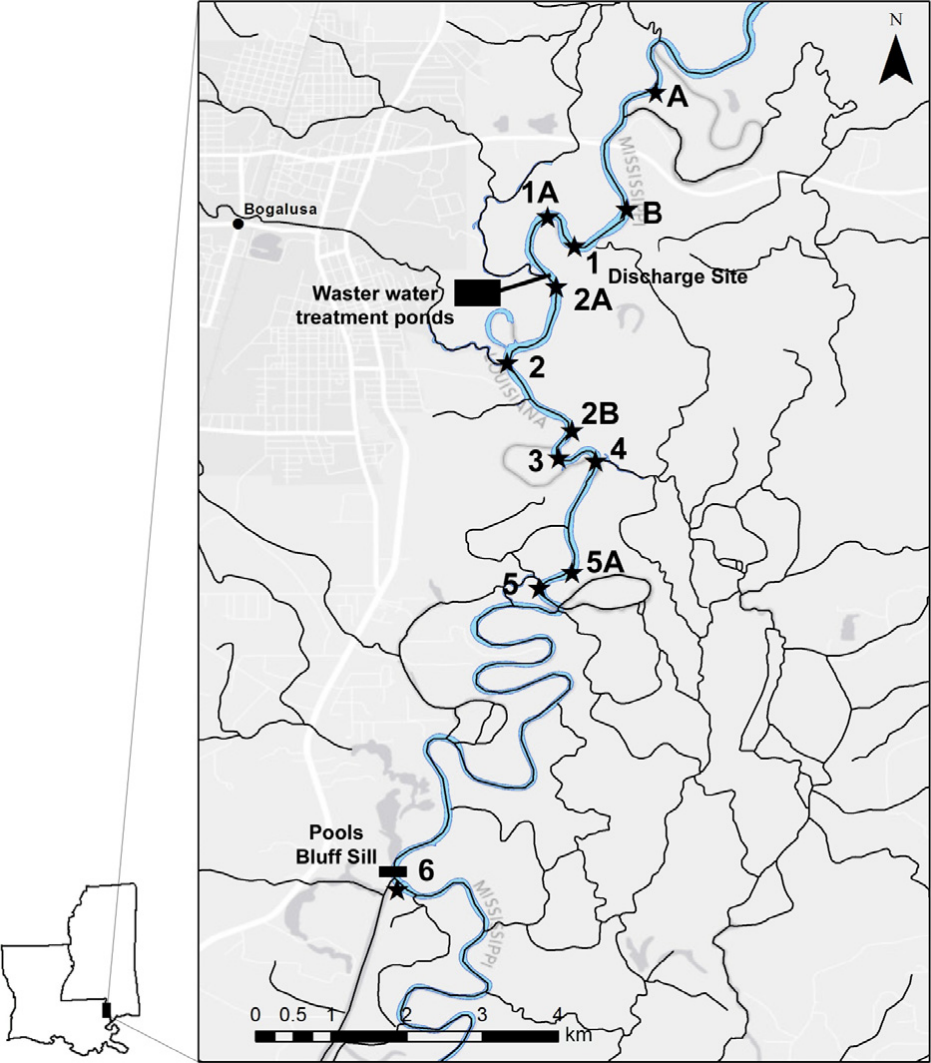
Distribution of the wastewater treatment ponds, discharge site, and fish sampling locations in the Pearl River, Louisiana, and Mississippi. Stations A, B, and 1–6 represent historic sampling localities, whereas 1A, 2A, 2B, and 5A represent sampling stations added to the postdischarge phase of the study.

Fishes were collected quarterly from eight sites (A, B, 1–6) in the Pearl River each year preceding the discharge event (Fig.2) (Geheber and Piller 2012). This included three sites above the discharge and five sites below the discharge. Sampling quarters typically correspond to the months of January, April, July, and October. Following the spill event in August 2011, four additional sites (1A, 2A, 2B, and 5A) were sampled alongside the original eight sites (i.e., a total of four sites above and eight sites below the discharge), and collections were made on a monthly basis (August 2011–December 2012); however, two sampling events were conducted in September 2011, and due to high water levels, no samples were taken in February 2012. The first field collections occurred approximately 2 weeks postdischarge. Sites were located along the shoreline on the inner bends of the river, and each site was approximately 100 m in length. Fishes were collected at each site using a 10-foot seine (10'× 6', 3/16” ace mesh) pulled in the downstream direction. Each site was sampled for approximately 15 min, and collection efforts were standardized following the historic sampling protocols initiated in 1988 (Geheber and Piller 2012). It should be noted that seining methods were standardized across sites and years making all samples comparable despite any potential size or taxonomic sampling bias associated with seining. Fishes collected were fixed in 10% commercial grade formaldehyde, and later transferred to 70% ethanol and permanently archived in the Southeastern Louisiana Vertebrate Collection. Fish collections from previous study years (1988–2010) were incorporated in later analyses examining long-term assemblage structure patterns (see Geheber and Piller 2012; K. R. Piller, unpubl. data). These historic collections used the same protocol as stated above (i.e., same eight sites sampled quarterly each year).

### Data analyses

Pearl River fish communities were examined at both fine temporal scales and broad temporal scales. Examining communities in this manner allowed us to observe local effects of the discharge event on a month-to-month basis (i.e., within 2011) and also observe the effects of the discharge event in a broader context (i.e., across years). This approach was necessary to properly assess any immediate impact that the discharge event had on the system and to determine the magnitude of any effects in relation to previous study years. Statistical analyses were performed using PRIMER 6 (Plymouth Routines In Multivariate Ecological Research) (2008 PRIMER-E Ltd: Lutton, Ivybridge, United Kingdom).

### Assemblage structure

Mean species richness and mean abundance per site values were reported for each sampling period during 2011 and 2012, and upstream of discharge and downstream of discharge values were calculated independently. Due to low total abundance values directly following the spill, we reported average species richness per site, within each sampling period (i.e., rarefaction was not practical due to the small sample sizes directly following the spill event). Assemblage data collected from the nine sampling periods during 2011 (i.e., January, April, July, August, September 1, September 2, October, November, and December) were used to examine fine temporal effects of the discharge event on assemblage structure. Only samples collected during 2011 were included in these analyses in order to remove unnecessary variation from multiple year comparisons. Sites were grouped a priori as either upstream of discharge (i.e., control group), or downstream of discharge (i.e., treatment group), within each of the nine sample periods. Data were square-root-transformed in order to buffer the effects of overly abundant species, and a permutational multivariate analysis of variance (PERMANOVA) was employed to test for differences in assemblage structure across sample periods and between areas (i.e., upstream and downstream sites) (Anderson 2001). Areas were treated as fixed effects, and sample periods were treated as random effects. Moreover, PERMANOVA was used due to its ability to partition response data according to sample design, and it includes interactions among factors. This was run using 9999 permutations based on Bray–Curtis similarity values calculated from the transformed dataset. Additionally, measures of multivariate dispersion (MVDISP) were used to determine the amount of variation in assemblage structure during each sampling period. This was useful in determining the magnitude of dissimilarity in assemblage structure (i.e., between upstream and downstream sites) during each sampling period. In order to visualize relationships among sample periods and upstream and downstream sites, data were averaged for each of the eighteen groups, and nonmetric multidimensional scaling (NMDS) based on Bray–Curtis similarity was run. The eighteen groups consisted of the upstream sites pooled across the nine sample periods, and the downstream sites pooled across the nine sample periods.

Similarity percentage (SIMPER) analysis was used to compare assemblage composition between upstream and downstream sites for the two sample periods following the discharge event. SIMPER provided average abundances of species within each area, and the standard deviation divided by the average dissimilarity of each species. Therefore, a species with a high average dissimilarity and a low standard deviation is a good contributor to the differences between areas. Species contributing to the majority of dissimilarity (>90%) were reported.

### Broad scale assemblage structure

Assemblage collections from 1988 to 2012 were averaged by year, and square-root-transformed to down weight the influence of overly abundant species. Nonmetric multidimensional scaling (NMDS) was run from a Bray–Curtis similarity resemblance matrix using 50 restarts and a minimum stress value of 0.01. Hierarchical complete linkage cluster analysis coupled with a similarity profile (Simprof) test of significance was run in addition to NMDS, and it was run from the same resemblance matrix. Simprof tested for genuine internal structure within the resultant dendrogram, and group significance was tested at *P* ≤ 0.05. Simprof is a priori unstructured and therefore allows comparisons between individual samples rather than between predefined groups (see Clarke et al. 2008 for further description).

## Results

### Assemblage structure

**Figure 3:**
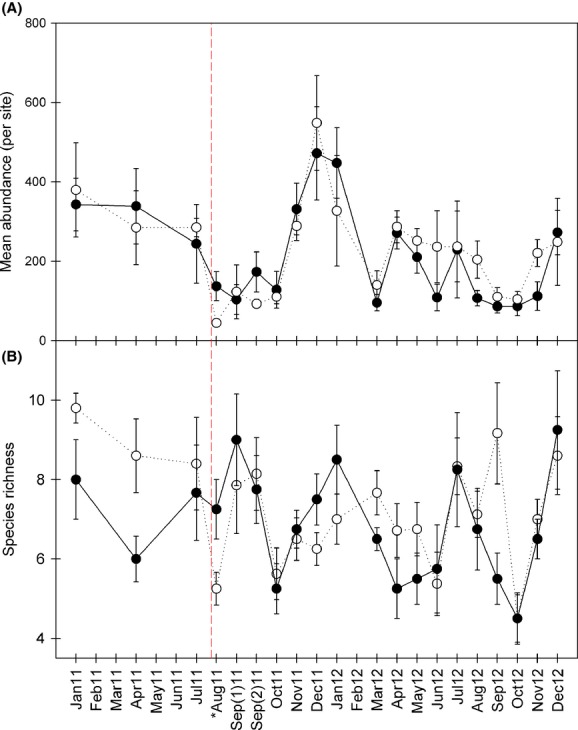
(A) Mean abundances (per site) across all sample periods during 2011 and 2012 (upstream and downstream of discharge areas are plotted separately). (B) Mean species richness (per site) within sample periods during 2011 and 2012 (upstream and downstream of discharge areas are plotted separately). Open circles represent below discharge sites, and filled (black) circles represent above discharge sites. The red vertical dashed line is the discharge event, and the asterisk represents the first sample month following the event. Three sites above the discharge point and five sites below the discharge point were sampled prior to the event. Following the spill event in August 2011, four additional sites were sampled alongside the original eight sites (i.e., a total of four sites above and eight sites below the discharge). All error bars are standard error.

Mean abundances per site generally show similar patterns between upstream (stations 1–2A) and downstream (stations 2–6) sites across 2011 and 2012 (Fig.3A). The lowest mean abundance per site was seen in sites downstream of the discharge in August 2011. Species richness was greater in the downstream section (stations 2–6) for most sample periods across 2011 and 2012; however, August 2011 downstream sites harbored much lower species richness than upstream sites (Fig.3B).

**Figure 4:**
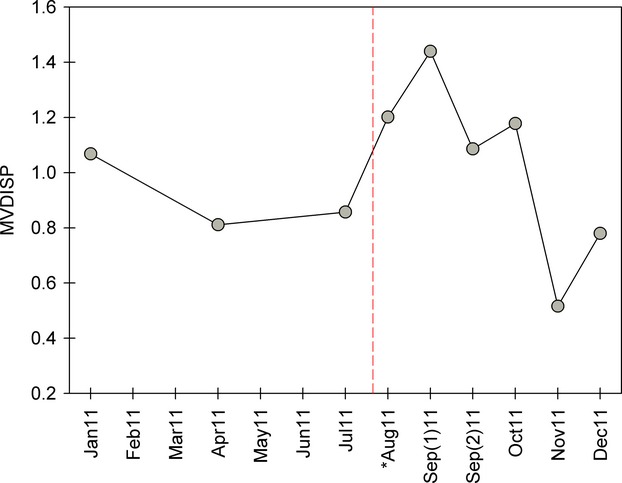
Multivariate dispersion (MVDISP), a measure of compositional variability, among all sites within each of the nine sampling periods during 2011. The asterisk indicates the first sample after the discharge event, and the vertical dashed line represents the event.

**Figure 5:**
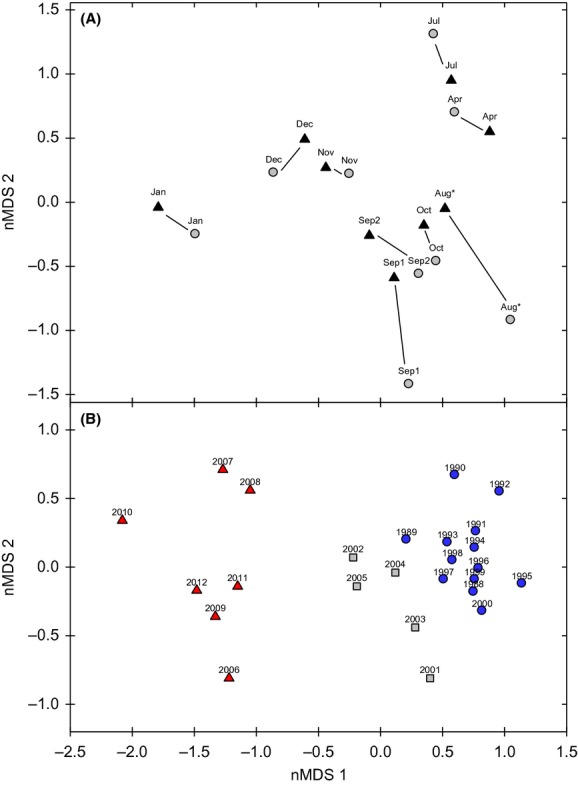
(A) Nonmetric multidimensional scaling (NMDS) plot depicting the relationship among the nine sample periods in 2011 (January–December). Black triangles are sites above the discharge. Gray circles are sites below the discharge. The asterisks indicate the month of the discharge event. The 2D stress value is 0.09. (B) Nonmetric multidimensional scaling of combined years (1988–2012). The 2D stress value is 0.09. Different colored groups represent significant structure recovered from the Simprof test (*P* ≤ 0.05) run with the accompanying cluster analysis.

Permutational multivariate analysis of similarity (PERMANOVA) reported significant sample period groups across area groups (df = 8, pseudo-*F* = 7.237, *P* < 0.001), and significant differences in areas across sample periods (df = 1, pseudo-*F* = 4.483, *P* = 0.008). However, the interaction between sample period and area was not significant (df = 8, pseudo-*F* = 1, *P* = 0.473). Multivariate dispersion (MVDISP) showed the greatest variances in assemblage structure during sample periods directly following the discharge event (Fig.4). This indicates greater differentiation among sites of upstream and downstream areas following the discharge event during August, September, and October sampling periods. These relationships were further illustrated in the NMDS which displayed seasonal relationships among the nine sample periods of 2011, as well as the area relationships (i.e., upstream and downstream) for each sample period (Fig.5A). Upstream and downstream sites were generally similar seasonally across sample periods (i.e., tightly paired) with the exception of the August sample period and the first sample period of September, which represent the 2 months of sampling postdischarge.

The SIMPER analysis resulted in eleven species which contributed to 90.86% of the dissimilarity between upstream and downstream areas during the two sample periods following the discharge event. The average dissimilarity between upstream and downstream sites was 52.87%, and eight of the eleven contributing species had greater average abundances in the upstream sites (Table1).

**Table 1:** SIMPER comparing community composition between sites above and below the discharge during the two sample periods following the discharge event (August and September 1) in 2011. Average dissimilarity between upstream and downstream areas = 52.87%

Species	Family	Above Discharge	Below Discharge	Avg. Diss	Diss./SD	Contrib. %	Cum. %
		Avg. Abundance	Avg. Abundance				
*Hybognathus nuchalis*	Cyprinidae	6.02	2.63	11.21	1.32	21.21	21.21
*Cyprinella venusta*	Cyprinidae	6.03	5.12	7.43	1.69	14.04	35.25
*Ammocrypta beani*	Percidae	2.40	0.16	6.26	1.52	11.83	47.09
*Gambusia affinis*	Poeciliidae	0.43	1.95	4.54	0.89	8.58	55.67
*Pimephales vigilax*	Cyprinidae	1.32	0.80	3.10	1.31	5.86	61.52
*Notropis longirostris*	Cyprinidae	1.85	1.59	3.04	1.24	5.75	67.28
*Notropis texanus*	Cyprinidae	1.25	0.56	3.03	1.10	5.74	73.02
*Lepomis megalotis*	Centrarchidae	0.70	0.96	2.77	1.18	5.24	78.26
*Dorosoma petenense*	Clupeidae	0.43	1.31	2.72	0.61	5.14	83.40
*Notropis atherinoides*	Cyprinidae	0.87	0.20	2.23	0.95	4.21	87.61
*Labidesthes sicculus*	Atherinopsidae	0.52	0.41	1.72	0.83	3.26	90.86

**Figure 6:**
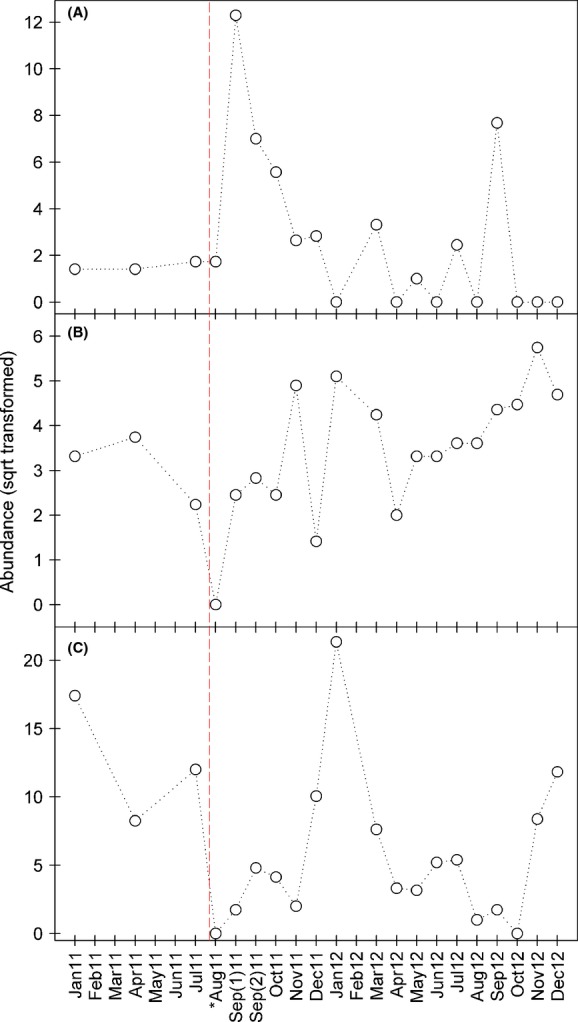
Abundances (per sample period) for three species from the Pearl River. (A) *Gambusia affinis* (Poeciliidae), (B) *Ammocrypta beani* (Percidae), and (C) *Notropis volucellus* (Cyprinidae) during each sampling period across 2011 and 2012 (i.e., sites below the discharge included). Abundance data were square-root-transformed. The vertical red dashed line indicates the discharge event. and the asterisk (*) indicates the first sample month following the event.

Additionally, Fig.6 shows abundances of three species below the discharge site across all 2011 and 2012 sampling periods. *Ammocrypta beani* (Percidae) and *Notropis volucellus* (Cyprinidae) each showed an absence in abundance directly following the discharge event, whereas *Gambusia affinis* (Poeciliidae) showed a rapid increase in abundance during sampling periods directly following the event (Fig.6).

### Broad scale assemblage structure

The NMDS for all sites combined across years (1988–2012) depicts assemblage structure relationships across the 25-year temporal scale (Fig.5B). Different colored year groups were found to be significantly different (*P* < 0.05) based on the simprof test from the accompanying cluster analysis. Groups were significantly similar at ≥65% Bray–Curtis similarity. Results show three significant year groups, where 2011 is not differentiated from chronologically close years (i.e., 2006, 2007, 2008, 2009, 2010, and 2012).

## Discussion

The role of disturbance and assemblage recovery has been studied for over a century, (Cowles 1899; Shelford 1911; Clements 1916; Gleason 1926; Odum 1969), but these seminal studies, and many since, primarily have been based on floristic models of succession and recolonization in a terrestrial setting. Assemblage recovery studies of animal communities, particularly in the aquatic realm, have received relatively less study (Resh et al. 1988; Detenbeck et al. 1992; Milner and Robertson 2010; Stanley et al. 2010; Matthews et al. 2013). Cairns (1990) noted that little was known about riverine ecosystems recovery following perturbations due to the lack of a “robust theoretical” framework for understanding these unique and dynamic systems. It is well accepted that fish assemblages are structured by a variety of intrinsic and extrinsic (i.e., biotic and abiotic) factors, (Strong 1983; Grossman et al. 1990; Matthews 1998), and in the absence of large-scale disturbance events, assemblages often maintain assemblage structure across time (Geheber and Piller 2012). However, disturbance events, especially human-caused disturbances, pose unique challenges (i.e., rarely or never encountered) for assemblages, which is concerning for the maintenance and/or recovery of assemblage structure in aquatic systems.

Theoretically, the degree and rate of assemblage recovery for any ecosystem should be proportional to the time frame and/or degree of intensity of the perturbation (Holling 1973). In some cases, assemblages are restructured following a natural or anthropogenic perturbation and never return to predisturbance conditions (Casselman et al. 1999; Pollard et al. 2003; Riley et al. 2008; Gido et al. 2010). In the case of terrestrial communities, recovery time is often gradual and substantial due to the time is takes for vegetation regrowth and subsequent recolonization by animals, whereas, in aquatic systems, particularly lotic environments, the longitudinal, downstream directional nature of the river current, the isolation of tributaries and backwater habitats from a main channel perturbation, and the short-generation time of most aquatic organisms can result in rapid recovery of an assemblage through recolonization from refugia. The period between the perturbation and recovery is often stochastic in terms of the recolonization of species and represents a period of instability, followed by recovery or stabilization. In this case, we feel that the “hangover effect” is an appropriate term applied to the recovery period of a short-term or pulse perturbation, as this time-lagged response mimics a hangover where short-term negative consequences are followed by a relatively rapid return to predisturbance conditions (Fig.1).

The major issue with an event such as this is “What constitutes recovery?” A return to predisturbance condition. Recovery to a general steady state or equilibrium assemblage. A completely novel and newly restructured assemblage. There does not seem to be a generalized assemblage response because all of the situations have been observed at one time or another under different conditions, durations, and intensities (see Matthews et al. 2013). As a result of the nature and duration of the Pearl River weak black liquor spill, a rapid return in community structure to predisturbance conditions was the observed trajectory in our study. Furthermore, the long-term fish assemblage dataset recently analyzed by Geheber and Piller (2012) served as the temporal and spatial baseline assemblage data and was used to make comparisons in terms of the abundance and diversity of species that recolonized the impacted area after the fish kill. The dataset represents the predisturbance “reference” assemblage, as Geheber and Piller (2012) indicated that the fish assemblage of the Pearl River in the impacted area has remained relatively stable over the past decade. Other studies have noted the importance of long-term monitoring and museum vouchering for understanding community dynamics after perturbation (Elliot 1990; Connell and Sousa 1983; Cody and Smallwood 1996; Piller et al. 2004).

In the case of the Pearl River, the perturbation was short and not pervasive, and lasted only a few days. The short-term, low oxygen event negatively impacted the fish assemblage, but the abiotic conditions returned to normal within a few days of the original perturbation. Periods of sustained hypoxia can impact fish populations in a variety of ways. Many fish species attempt to survive via increased gill ventilation rates or through surface respiration, whereas others are capable of behaviorally avoiding the hypoxic area (Breitburg 2002). Gammon and Reidy (1981) noted that during a period of low oxygen in the Wabash River, Indiana, many species congregated near the outflows of tributaries, apparently in an effort to obtain oxygen. In other cases, many species are unable to avoid unfavorable oxygen conditions and inevitably perish.

The results from this present study indicate both temporal (i.e., month) and spatial differences in community structure across the 2011 sample year based on PERMANOVA. These differences are typical of seasonal variation in community structure within systems, and this was expected due to temporal variation previously found in the system across previous years (Geheber and Piller 2012). However, we were interested in testing for differences between upstream–downstream pairs prior to and following the discharge event. Because we expected seasonal variation to be correlated among all sites (i.e., in the absence of disturbance), our design allowed us to test for an interaction between upstream and downstream sites following the discharge event. However, we did not detect a significant interaction between site location (i.e., upstream and downstream) and sample period following the event, despite seeing a separation between upstream and downstream community structure in August and September 2011 (Fig.5A). The lack of significance suggested that the effects of the effluent spill were minimal on the fish community, and no significant change was found in effluent exposed site community structure directly after the event. Furthermore, no long-term effects (i.e., throughout 2011 samples) on community structure were detected in exposed sites, indicating that any recovery which may have been necessary for the return to predisturbance conditions occurred rapidly. In addition, we show a clear, rapid recovery to predischarge fish species richness and abundance values within 2 months following the perturbation. This was also shown by the sudden increase in MVDISP directly following the event, followed by a rapid return to previous, before spill, MVDISP values across upstream and downstream sites. This was representative of the amount of variation among upstream (control) sites and downstream (treatment) sites prior to, and following the discharge event. In the case of the Pearl River, the hangover effect only lasted for a short time frame. In fact, other studies have noted relatively rapid recolonization and recovery of fishes following stream defaunation events (Peterson and Bayley 1993; Sheldon and Meffe 1994) or low oxygen conditions (Knott and Martore 1991; Stevens et al. 2006). Jones & Schmitz (2009) conducted a meta-analysis of more than 200 studies of ecosystem recovery and noted that the recovery time of aquatic systems trended toward shorter recovery times relative to terrestrial ecosystems.

The weak black liquor release negatively impacted fish species abundances, and richness in the downstream reaches more substantially directly following the event (i.e., August 2011) (Fig.3). This was seen for downstream species richness and downstream average abundance, each of which was much lower than upstream control sites during the sampling period of August 2011 directly following the discharge event. Recovery of the fish assemblage is not totally unexpected considering the physical habitat was unimpacted and the abiotic parameters returned to normal levels within only a few days of the cessation of weak black liquor release. Upstream reaches of the Pearl River and its tributaries were not impacted by the low dissolved oxygen conditions and likely served as refugia and recolonization sources. Shortly after the abiotic conditions returned to normal, fishes may have repopulated the impacted area relatively quickly via the downstream currents.

Over the past fifty years, benthic fishes have been disproportionately impacted in the Pearl River as a result of substantial geomorphic instability in the Pearl River, resulting in significant declines in species richness and abundances (Piller et al. 2004; Tipton et al. 2004; Geheber and Piller 2012). In this study, the SIMPER analysis indicated that three species contributed >10% to the average dissimilarity among areas (above vs. below discharge). Two of these species were previously classified as nonbenthic generalist species by Piller et al. (2004), *Hybognathus nuchalis* (Cyprinidae) and *Cyprinella venusta* (Cyprinidae), and the other as a benthic-sand species, *Ammocrypta beani* (Percidae). Prior to the perturbation event, these two cyprinid species were typically the most abundant species collected in the survey area over the last 5 years, and the naked sand darter represents the most common darter species in the main channel of the Pearl River over the same time frame. Therefore, the rapid recovery of these species in the impacted area is not surprising. However, *A. beani* was absent from collections downstream of the discharge directly following the event (Fig.6). Furthermore, *Cyprinella venusta* is the most abundant, widespread, and generalistic cyprinid species along the entire Gulf Coastal Plain. Eight additional species contributed more than 3% to the average dissimilarity and all were nonbenthic generalist species.

The western mosquitofish (*Gambusia affinis*) was one of the most commonly collected species in the area downstream area following the perturbation (Fig.6). This species was abundant during the 2-month period following the perturbation, but was not abundant in the upstream reaches nor during the historical sampling periods at any of these same localities during the 22-year sampling period (Geheber and Piller 2012). Western mosquitofish are an aggressive generalist species that has previously been shown to be tolerant of poor environmental conditions, including high water temperatures and low dissolved oxygen conditions (Moyle 1976). Therefore, the appearance of western mosquito fish in the first few sampling periods following the perturbation is not surprising. Their dorsally oriented terminal mouths and flattened heads, as well as their preference for surface habitats (Lewis 1970; Cech et al. 1985), may have allowed them to utilize oxygen at the atmosphere–water interface during periods of low dissolved oxygen in the Pearl and persist where other species perished. Alternatively, western mosquitofish could have repopulated the area from the lower reaches of tributaries or vegetated backwater habitats of the Pearl River, the preferential habitat of the species (Ross 2001).

The loss of sportfishes (i.e., centrarchids and ictalurids) in the impacted area of the Pearl River is a great concern for local sportsman. In 2005, Hurricanes Rita and Katrina resulted in widespread fish kills for many sportfish species in the nearby Atchafalaya Basin in Louisiana and resulted in declines in catch-per-unit effort for many species of centrarchids for nearly 2 years following these events (Perrett et al. 2010). Based on the historical and contemporary sampling approach of our study, little information can be ascertained on the loss and recovery of large bodied fishes (i.e., *Ictiobus* spp., *Ictalurus* spp.) in the impacted area of the Pearl River because the sampling gear utilized in this present study occasionally captures juveniles and subadults in small numbers and rarely captures adults from the sand bar habitats. Alternative sets of sampling gears will need to be employed to quantitatively assess the long-term impacts on the sport fish community. However, it will likely take many years for the biomass of commercial and recreational fisheries to recover in the basin, similar to what previously occurred in the Atchafalaya Basin, but this is being expedited by substantial stocking efforts of ictalurids and centrarchids by state natural resource agencies in the Pearl River downstream of the discharge site.

## Summary

The Pearl River fish assemblage is resilient, and the rapid recovery of the assemblage to preperturbation conditions is a testament to that and several mechanisms likely contributed to the rapid recovery. First, specific life-history traits, including a species' dispersal or migratory abilities, likely played a role in the recolonization of the impacted areas. Unfortunately, examining an individual species migratory ability and maximum daily dispersal distances is challenging and, as a result, this information remains unknown for many species. However, previous studies have noted rapid rates of recolonization, from hours to weeks, following a disturbance or defaunation event (Matthews et al. 1987; Peterson and Bayley 1993; Sheldon and Meffe 1994) suggesting that some stream species are capable of substantial dispersal and colonization movements in a short period of time.

Second, the availability and proximity of tributaries and upstream refugia likely played a substantial role in the fish assemblage's recovery to predischarge conditions within only a few months. Although stream fishes are believed to be sedentary and have relatively small home ranges (Gerking 1953, 1959; Gowan et al. 1994; Rodríguez 2002), a variety of factors may facilitate movement and an increase in home ranges including the availability of habitat and food resources. In the case of the Pearl River, upstream areas were not impacted by the release of the weak black liquor, and the short-term, low oxygen conditions. Therefore, an increase in home ranges as a result of reduced competition for habitat and recolonization by macroinvertebrates may also have played a role in rapid recolonization of fishes from upstream habitats in the Pearl River.

Finally, other studies have investigated the challenges of fish dispersal from both biotic and abiotic perspectives. Other factors including the morphology of the stream, current velocities, and the abundance of predators can play a role in the dispersal ability of a particular species, and different species can respond differently to these factors (Schaefer 2001). As a result, recolonization of the fish assemblage, although rapid, takes time to return to predisturbance assemblage values.

The knowledge of long-term fish assemblage dynamics within the Pearl River is invaluable (Piller et al. 2004; Geheber and Piller 2012). These data allow for more meaningful and informative pre- and postdisturbance comparisons to be made and allow for a more informed understanding of the processes of disturbance and recovery. Without dedicated monitoring efforts and the vouchering of specimens, interpretation of fish assemblage recovery would be challenging.
